# Integrated QTL mapping and candidate gene analysis for yield-related traits and salt tolerance in a rice RIL population

**DOI:** 10.3389/fpls.2025.1711018

**Published:** 2026-01-20

**Authors:** Yanhong Zhang, Yulong Wang, Xiaojing Du, Xiaorong Wen, Mintai Kang, Tianyu Hou, Fusen Tang, Yuhong Qi, Zhiqiang Zhao, Quan Yuan, Abliz Bhaliqem, Dong Li, Fengbin Wang, Jie Yuan

**Affiliations:** 1 Crop Research Institute of Xinjiang Uygur Autonomous Region Academy of Agricultural Sciences/Northwest Center of National Salinity Tolerant Rice Technology Innovation Center, Urumqi, Xinjiang, China; 2 Agricultural College, Henan University of Science and Technology, Luoyang, Henan, China

**Keywords:** rice, yield-related traits, salt, QTL mapping, molecular breeding

## Abstract

Rice is a globally critical staple crop, and enhancing its yield and stress resilience is essential for food security. In this study, we employed a recombinant inbred line (RIL) population derived from cultivars Liangxiang5 and 03GY28 to dissect the genetic basis of yield-related traits, leaf color, and germination stage salt tolerance. A high-density genetic map was constructed using 1, 101 bin markers, spanning 1, 132.95 cM with an average marker interval of 1.03 cM. Over two-year field trials, we identified 16 quantitative trait loci (QTLs) for nine agronomic traits distributed across chromosomes 3, 5, 6, 7, 9, and 11. These QTLs accounted for 5.48%-19.03% of phenotypic variance (PVE), with LOD scores ranging from 2.52 to 8.93. Notably, a major-effect QTL, qLeafColor9.1, explaining 19.03% of variance and was mapped to chromosome 9 and co-localized with the known senescence-associated gene *OsSGR*, which was significantly upregulated under salt stress. Additionally, QTL hotspots on chromosomes 9 and 11 governed multiple yield-related traits-including panicle branching number, filled grain number, and total grain number-including pleiotropy or tight gene linkages. Additionally, two salt tolerance-related QTLs (qRSDR6.1 and qRSDR7.1) were identified, and candidate genes responsive to abiotic stress were annotated within these intervals. These findings advance our understanding of the genetic architecture underlying rice agronomic traits and provide actionable targets for marker-assisted breeding to improve yield, stress tolerance, and grain quality.

## 1 Introduction

Rice (*Oryza sativa* L.) is one of the most important staple crops globally, serving as a primary food source for over half of the world’s population (Guo et al., 2025; Ma X. et al., 2025). With the increasing challenges posed by climate change, population expansion, and diminishing arable land, improving rice yield and stress tolerance has become a central goal in modern breeding programs. Grain yield (Ma Z. et al., 2025; Yang et al., 2025), leaf color (Yuan et al., 2025), and germination stage salt stress tolerance (Li Y. et al., 2022) are key agronomic traits. They are especially significant because they directly influence productivity, resource-use efficiency, and overall crop resilience.

Grain yield is a complex quantitative trait controlled by multiple genes and significantly influenced by environmental conditions. Over the past decades, numerous quantitative trait loci (QTLs) associated with yield components such as panicle number, grain number per panicle, and grain weight have been identified in rice (Yun et al., 2024; Zhao H. et al., 2024; Zhao X. et al., 2024). However, the genetic basis of yield variation remains only partially elucidated, primarily due to the polygenic control and significant genotype-by-environment interactions. Thus, dissecting the genetic architecture of grain yield through high-resolution mapping populations is essential for marker-assisted selection and facilitating gene cloning efforts.

Leaf color, often associated with chlorophyll content and photosynthetic efficiency, is not only an indicator of plant health but also a key determinant of biomass accumulation and yield potential (Qiu et al., 2024). Variations in leaf coloration often reflect underlying differences in nutrient uptake, light utilization efficiency, and adaptive responses to environmental stresses. Although several genes involved in chlorophyll biosynthesis and degradation have been cloned, the genetic control of natural variation in leaf color, especially under complex field conditions, is still not well understood (Shin et al., 2020; Xie et al., 2025). A deeper understanding of the genetic mechanisms regulating leaf color could contribute to the development of rice varieties with improved photosynthetic performance and stress adaptability.

Salt stress is one of the most severe abiotic stresses limiting global rice production and the expansion of arable land (Wu et al., 2025). It is particularly detrimental during the seed germination stage, which is highly vulnerable to salinity. The ability of seeds to successfully germinate and establish robust seedlings in saline soils directly determines the seedling establishment rate, population uniformity, and ultimately the final yield in direct-seeding rice systems. Therefore, enhancing salt tolerance at the germination stage is of paramount importance for ensuring food security.

Conventional breeding efforts have been hindered by the polygenic nature of salt tolerance and the scarcity of efficient, scalable phenotyping protocols. However, recent progress in genomics and high-throughput phenotyping has accelerated the discovery of genetic loci associated with this trait. Using both linkage analysis and association mapping, several QTLs conferring stable germination-stage salt tolerance across diverse environments have been identified (Lin et al., 2004; Li et al., 2021; Goto et al., 2022; Geng et al., 2024; Wei et al., 2024; Xu et al., 2024). Furthermore, a series of key genes have been successfully cloned, including the Na^+^ transporter gene *OsHKT1;5* (Ren et al., 2005; Wang et al., 2020), the cytokinin signaling gene *OsRR22* (Liu et al., 2023), and the transcription factors *OsGRF7* (Chen et al., 2024) and *OsWRKY53* (Yu et al., 2023). These genes enhance salt tolerance by regulating ion homeostasis, root system development, and stress-responsive transcriptional networks, respectively.

These findings have greatly advanced our understanding of the molecular basis of salt tolerance and have offered functional gene targets for molecular breeding. Nevertheless, the number of salt tolerance genes that have been successfully deployed in breeding programs and demonstrated significant phenotypic effects remains limited. The homozygosity and genetic stability of RIL populations, combined with high-density resequencing, provide a powerful foundation for high-resolution QTL mapping and candidate gene identification (Li F. et al., 2024; Wang F. et al., 2024; Hou et al., 2025; Li Z. et al., 2025; Shi et al., 2025; Wang et al., 2025). This study was designed to address the genetic sources of the pronounced phenotypic divergence between Liangxiang5 and 03GY28 in key agronomic traits. We specifically sought to: construct a high-density bin map from a resequenced RIL population; systematically map QTLs for yield-related traits, leaf color, and salt tolerance; and evaluate the potential of identified favorable alleles for marker-assisted selection.

## 2 Materials and methods

### 2.1 Plant materials

A RIL population consisting of 207 lines was developed from a cross between the rice cultivars Liangxiang5 (female parent) and 03GY28 (male parent). The population was advanced through eight generations of continuous selfing using the single-seed descent (SSD) method. Significant phenotypic differences between the two parental lines were observed for multiple agronomic traits, including: primary branch number (PBN), filled grain number per panicle (FGN), total grain number per panicle (TGN), 1000-grain weight (TGW), length/width ratio (LWR), mean grain length (MGL), mean grain width (MGW), leaf color, relative salt damage rate (RSDR).

### 2.2 Phenotypic data collection and statistical analysis

A field trial was conducted at the experimental station of the Wensu Rice Experimental Station, Crop Research Institute of Xinjiang Uygur Autonomous Region Academy of Agricultural Sciences during the 2020–2021 growing seasons (41°16’ N, 80°12’ E). The region is characterized by an average annual sunshine duration of 2800–3000 hours, an average annual precipitation of 75 mm, the mean annual evapotranspiration is 1, 200-1, 500 mm. and a frost-free period ranging from 200 to 220 days. It experiences cold winters and hot summers, with a mean annual temperature of 10.0 °C. The soil at the experimental site is clayey, with the following physicochemical properties (0–30 cm depth): pH 8.0, electrical conductivity 2.48 mS·cm^−^¹, organic matter 35.90 g·kg^−^¹, total nitrogen 2.29 g·kg^−^¹, available nitrogen 168.50 mg·kg^−^¹, available phosphorus 48.00 mg·kg^−^¹, and available potassium 156.00 mg·kg^−^¹. A total of 207 RILs along with their parental lines were planted using a completely randomized block design (CRBD) with three replicates. Seedlings were raised on April 2 and manually transplanted on May 9. Each variety planted in two rows with row measuring 2.00 m in length. Plant spacing was 15 cm × 30 cm. Border rows were established around the experimental plots to minimize edge effects. Field management practices were consistent with those applied in general production fields.

Phenotypic traits of the RIL population and parental lines were recorded at the maturity stage to ensure data consistency and comparability. All measurements were performed using standardized protocols. With reference to the Descriptors and Data Standard for Rice Germplasm (Han and Wei, 2006), seven quantitative traits were investigated, including PBN, FGN, TGN, TGW, LWR, MGL, and MGW. The stay-green trait was evaluated at the mature stage based on visual observation of the whole plant. Based on the criterion of leaf color retention, plants were designated as “green” if they maintained a deep green color, or “yellow” if they displayed yellow or light green coloration.

### 2.3 Salt stress treatment at germination stage

Fifty plump and uniform seeds of each accession were selected, surface-sterilized with 75% ethanol for 15 minutes, and rinsed three times with sterile water. The seeds were then placed in Petri dishes lined with filter paper. Two treatments were established: a control group (distilled water) and a salt stress treatment group (NaCl solution), with three replicates per treatment. For the treatment group, 15 mL of 200 mM sodium chloride (NaCl) solution was added to each dish. All dishes were covered and placed in a constant-temperature incubator at 30 °C for germination. Seed germination was observed daily, and the solution and salt concentration were maintained throughout the experiment. On the 7th day, the germination rate of each material was recorded. Salt tolerance at the germination stage was evaluated based on the relative salt damage rate:

Relative Salt Injury Rate (%) = (Germination Rate of Control – Germination Rate of Treatment)/Germination Rate of Control × 100

The collected data were initially recorded and processed using Microsoft Excel 2019. Descriptive statistics, correlation analysis, and frequency distribution histograms were generated using the R software (version 4.0).

### 2.4 RNA sequencing

When the seedlings of Nipponbare reached the three-leaf stage, Nipponbare seedlings having similar growth vigor were selected and treated for 0 h, 12 h and 24 h with 200 mM NaCl. The leaves were clipped and collected for transcriptome at the corresponding treatment time. Three biological replicates were performed for each sample, the raw transcriptome sequencing data is sourced from NCBI (PRJNA1037192).

### 2.5 DNA extraction and sequencing

At the tillering stage, young leaves were collected from the parents and progeny of the RIL population. The leaf samples were immediately frozen in liquid nitrogen and stored at −80 °C until DNA extraction. Total genomic DNA was extracted from each sample using a Plant DNA Extraction Kit, following the manufacturer’s instructions. The concentration and purity of the extracted DNA were measured using a NanoDrop 2000 spectrophotometer. Only DNA samples with an OD260/280 ratio between 1.8 and 2.2, a concentration of at least 40 ng/μL, and a total amount of no less than 2 μg were used for subsequent library construction.

DNA libraries were prepared using the TruSeq Library Construction Kit according to the standard protocol. The qualified libraries were then sequenced on the Illumina HiSeq platform to generate 150 bp paired-end reads.

The whole-genome re-sequencing of the parents and the RIL population and RNA-seq were performed by Novogene Bioinformatics Technology Co., Ltd. (Tianjin, China).

### 2.6 Detection and annotation of genome variation

Prior to alignment, the raw sequencing reads were filtered to remove low-quality reads containing ≥10% unidentified bases (N’s) or >50% of bases with a Phred quality score < 5 (Bolger et al., 2014). The cleaned reads were then aligned to the Nipponbare reference genome from the Rice Genome Annotation Project (MSU7, https://rice.uga.edu/download_osa1r7.shtml) using BWA (version 0.7.15-r1140) (Li and Durbin, 2009). The resulting alignment files were converted to BAM format using SAMtools (version 1.3.1) (Li et al., 2009). Only uniquely mapped reads were retained for subsequent SNP detection.

SNPs and InDels were identified across all samples using the Genome Analysis Toolkit (GATK, version 4.0) (McKenna et al., 2010). Candidate SNPs that were homozygous in both parents and the RIL population were selected for further analysis. To ensure high-quality variant calls, the following filtering criteria were applied: genotype missing rate ≤ 50%, heterozygosity rate ≤ 15%, and minor allele frequency (MAF) ≥ 20%. Finally, functional annotation of the variant sites was performed using SnpEFF (Cingolani et al., 2012).

### 2.7 Genetic linkage map construction based on bin map

A chi-square test was performed to assess the segregation ratio of markers. Markers showing significant segregation distortion (*P* < 0.001) were excluded from subsequent analyses. Genotyping was conducted using a sliding window approach with a window size of 15 consecutive non-missing SNPs. Within each window, if the number of SNPs originating from either parent was ≥11, the individual was assigned a homozygous genotype; otherwise, it was classified as heterozygous. Consecutive 100 kb genomic intervals with identical genotypes across the entire RIL population were grouped into a single recombination bin. These bins were utilized as genetic markers to construct a genetic linkage map using JoinMap 4.0 software (Ooijen, 2006), with a recombination frequency threshold of <0.4 and a minimum LOD (logarithm of odds) score of 6.

The integrated phenotypic and genotypic datasets were subjected to QTL mapping analysis using the inclusive Composite Interval Mapping (ICIM) method implemented in QTL IciMapping v4.2 (Zhang et al., 2022), with the Grouping module set to LOD > 2.5. QTL analysis was performed using inclusive composite interval mapping (ICIM) with a step size of 1.0 cM and a probability threshold for entering variables (PIN) set at 0.001. Genetic distances were calculated using the Kosambi mapping function, and the genetic linkage map was visualized using R software (version 4.0). A LOD threshold of ≥2.5 was used to declare significant QTLs for each trait. Based on the QTL mapping results, candidate genes within the QTL regions were annotated using rice genome functional information, and expression data were integrated to prioritize potential candidate genes. QTL nomenclature followed the McCouch (Mccouch et al., 1997) naming convention.

## 3 Results

### 3.1 Phenotypic variation of the parents and RIL individuals

From 2020 to 2021, a two-year field investigation was conducted to evaluate the phenotypic performance of seven yield-related traits in the parental lines and a RIL population (**Table 1**). The parental lines exhibited distinct differences in these traits. Overall, Liangxiang5 demonstrated superiority in grains per panicle, grain length, and length-to-width ratio, whereas 03GY28 possessed higher thousand-grain weight and grain width (**Supplementary Table S1**).

**Table 1:** Phenotypic statistics of seed traits in parental and RIL populations across two environments.

TraitID	Environment	Number	Minimum	Maximum	Range	Mean	SD	Skewness	Kurtosis	CV(%)
PBN	2020	207	5.67	10.56	4.89	7.82	0.97	0.11	-0.50	12.43
	2021	198	6.56	11.89	5.33	9.43	0.93	-0.04	-0.05	9.85
	Average	198	6.94	10.50	3.56	8.62	0.73	0.02	-0.30	8.44
FGN	2020	207	35.44	136.44	101.00	82.75	17.42	0.10	0.06	21.06
	2021	198	57.44	127.33	69.89	98.29	13.17	-0.81	0.34	13.40
	Average	207	55.11	124.33	69.22	90.17	12.82	-0.02	0.18	14.22
TGN	2020	207	54.44	148.22	93.78	93.42	17.73	0.23	-0.13	18.98
	2021	207	53.12	146.89	93.77	92.25	17.85	0.24	-0.16	19.35
	Average	207	53.78	147.56	93.78	92.84	17.78	0.23	-0.15	19.15
TGW	2020	205	22.20	37.44	15.25	27.86	2.97	0.43	-0.10	10.67
	2021	199	19.65	34.46	14.81	26.58	2.98	0.30	-0.54	11.20
	Average	199	20.92	34.69	13.77	27.20	2.81	0.35	-0.42	10.34
LWR	2020	205	2.15	3.67	1.53	2.77	0.27	0.20	-0.32	9.84
	2021	199	1.81	3.65	1.85	2.77	0.32	0.01	-0.37	11.69
	Average	207	2.15	3.60	1.45	2.76	0.28	0.11	-0.53	10.25
MGL	2020	205	4.99	9.75	4.77	7.58	1.13	-0.50	-0.88	14.95
	2021	199	4.93	9.84	4.91	7.64	1.27	-0.52	-0.91	16.66
	Average	207	4.99	9.80	4.81	7.59	1.20	-0.51	-0.98	15.80
MGW	2020	205	1.85	3.76	1.90	2.74	0.37	-0.47	-0.53	13.57
	2021	200	1.74	3.46	1.72	2.77	0.42	-0.64	-0.74	15.07
	Average	207	1.81	3.61	1.80	2.75	0.39	-0.56	-0.75	14.23
RSDR	2021	207	7.07	100.00	92.93	71.67	20.07	-0.82	0.21	28.00

In 2020, leaf color at the maturity stage was recorded. Liangxiang5 exhibited yellow-green leaves, whereas 03GY28 maintained dark green leaves. In 2021, germination rates under salt stress were assessed for both parents and the RIL population. The relative salt damage rate (RSDR) of 03GY28 was 35.66%, while that of Liangxiang5 was 84.03%.

**Figure 1:**
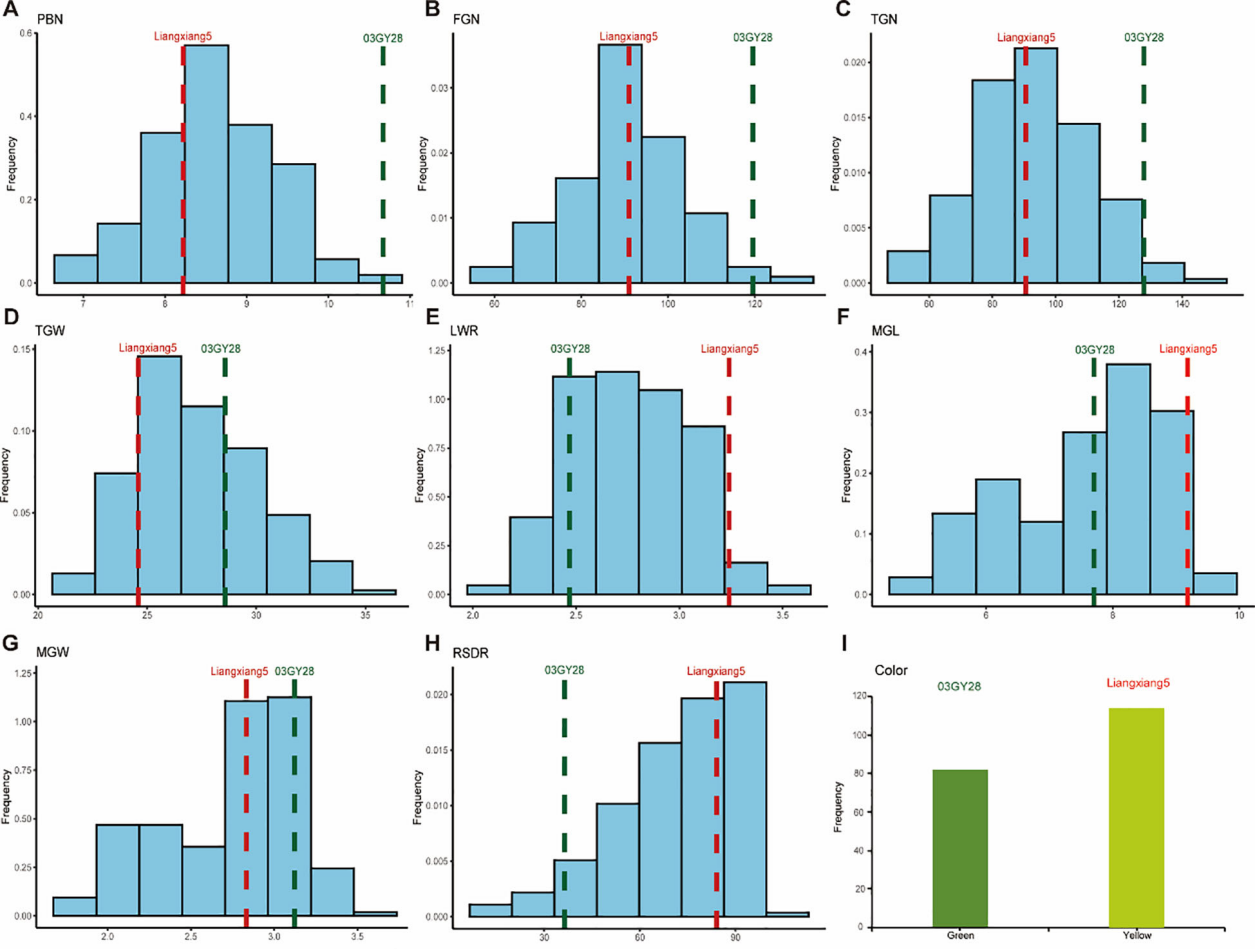
Phenotypic variation of RIL populations and parents. **(A)** primary branch number (PBN). **(B)** filled grain number (FGN). **(C)** total grain number (TGN). **(D)** 1000-grain weight (TGW, g). **(E)** length/width ratio (LWR). **(F)** mean grain length (MGL, mm). **(G)** mean grain width (MGW, mm). **(H)** relative salt damage rate (RSDR). **(I)** leaf color.

Both yield traits and RSDR in the RIL population showed significant phenotypic variation with continuous distributions (**Figures 1A-H**), as evidenced by broad value ranges and moderate to high coefficients of variation (CV%) (**Table 1**). These results suggest that the traits are quantitatively inherited. FGN displayed the widest variation range (69.22–101.00) and the highest CV% (13.40–21.06), indicating significant segregation and considerable potential for genetic improvement. In comparison, LWR and PBN showed relatively low CV% (8.44–11.69), suggesting higher stability across different environments. The mean values of all traits in the RIL population fell between those of the two parents, while the minimum and maximum values exceeded the parental ranges. The absolute values of skewness and kurtosis for all traits were less than 1, indicating that these traits followed an approximately normal distribution. Additionally, significant segregation in leaf color at maturity was also observed within the RIL population (**Figure 1I**).

### 3.2 Correlation analysis

**Figure 2:**
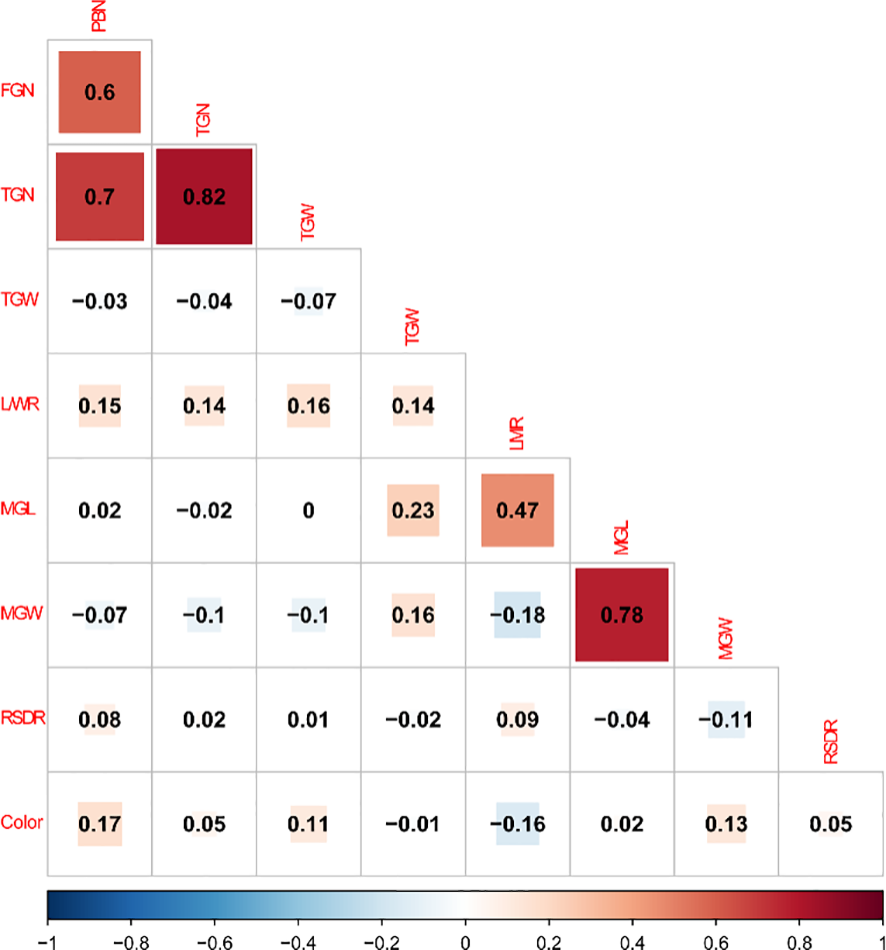
Correlation analysis of nine traits in RIL population.

Correlation analysis revealed significant relationships among key agronomic traits in the rice RIL population (**Figure 2**, **Table 2**). The strongest positive correlation was observed between TGN and FGN (r = 0.91), indicating that genotypes with higher total grain production tend to produce more filled grains. TGW was also moderately correlated with FGN (r = 0.59) and TGN (r = 0.35), suggesting a synergistic effect between grain yield components. Notably, most morphological traits, such as LWR, MGL and MGW, exhibited weak or non-significant correlations with yield-related traits, highlighting their potential as independent indicators for grain quality evaluation. These findings provide valuable insights into trait interactions and support future QTL mapping and marker-assisted selection for rice improvement.

**Table 2:** Statistical significance of phenotypic trait correlations.

Trait	PBN	FGN	TGN	TGW	LWR	MGL	MGW	RSDR	Color
PBN	NA	0	0	0.7	0.03	0.8	0.33	0.26	0.02
FGN	0	NA	0	0.59	0.05	0.73	0.16	0.77	0.47
TGN	0	0	NA	0.35	0.02	0.96	0.17	0.91	0.11
TGW	0.7	0.59	0.35	NA	0.06	0	0.02	0.75	0.87
LWR	0.03	0.05	0.02	0.06	NA	0	0.01	0.18	0.03
MGL	0.8	0.73	0.96	0	0	NA	0	0.61	0.77
MGW	0.33	0.16	0.17	0.02	0.01	0	NA	0.1	0.08
RSDR	0.26	0.77	0.91	0.75	0.18	0.61	0.1	NA	0.49
Color	0.02	0.47	0.11	0.87	0.03	0.77	0.08	0.49	NA

### 3.3 Sequencing data analysis and construction of genetic map

To construct a high-density genetic linkage map in rice, whole-genome resequencing was performed on 207 F_6_ individuals and their parental lines. The parental lines, Liangxiang5 and 03GY28, generated 7.76 Gb and 8.46 Gb of clean reads, respectively (**Supplementary Table S2**). A total of 741.77 Gb of clean reads were obtained from the 207 RILs. After aligning the clean reads to the reference genome, the parental alignment rates exceeded 99.80%, with average coverage depths greater than 16.53X, 1X coverage above 97.02%, and 4X coverage above 93.55%. For the progeny, the average alignment rate was 99.79%, with an average coverage depth of 7.41X and mean 1X coverage over 96.14% (**Supplementary Table S3**).

**Figure 3:**
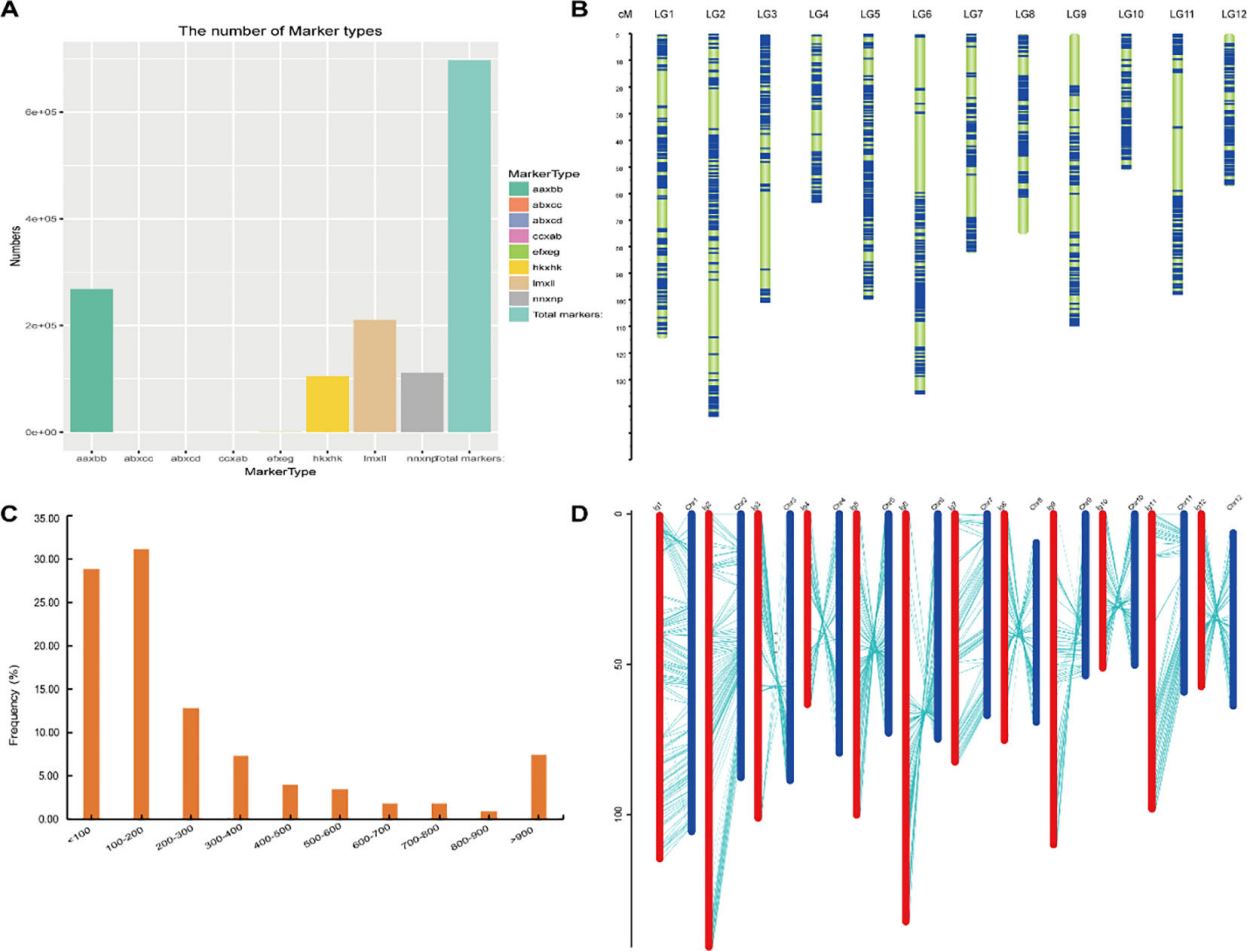
Marker screening and genetic map construction. **(A)** The number of Marker types. **(B)** Map of marker distribution in linkage groups. **(C)** Distribution of bin lengths. **(D)** Collinearity between genetic map and physical map.

Based on the parental genotyping results, polymorphic markers between the parents were developed. After filtering out loci with missing parental information, a total of 697, 294 polymorphic loci were identified (**Supplementary Table S4**). Among these, 268, 279 homozygous SNPs (“aaxbb” type) were detected between the two parents (**Figure 3A**). Following standard genotyping filtering in the progeny, 60, 100 SNPs were retained for bin marker generation in the F_6_ population. SNPs showing significant segregation distortion (*P* < 0.001) were excluded from subsequent analyses.

An improved sliding window approach was employed to identify recombination breakpoints in the RILs. Adjacent bins with identical genotypes were merged into a single recombination bin, resulting in the construction of a high-density genetic map comprising 1, 101 recombination bin markers (**Figure 3B**). The cumulative physical length of all bins was 365 Mb, with individual bin sizes ranging from 580 bp to 5.33 Mb and an average size of 331.72 kb (**Figure 3C**). A total of 12 linkage groups were generated. The total genetic distance of the bin map was 1132.95 cM, with an average distance of 1.03 cM between adjacent bin markers (**Supplementary Table S5**). Notably, 97.28% of the intervals between adjacent bin markers were less than 5 cM (**Supplementary Table S6**). Collinearity analysis was performed by comparing the physical positions of the markers on the genome with their genetic locations on the linkage map (**Figure 3D**). The high degree of collinearity observed indicates that the marker order is accurate and the map is of high quality.

### 3.4 QTL mapping for eight traits

**Figure 4:**
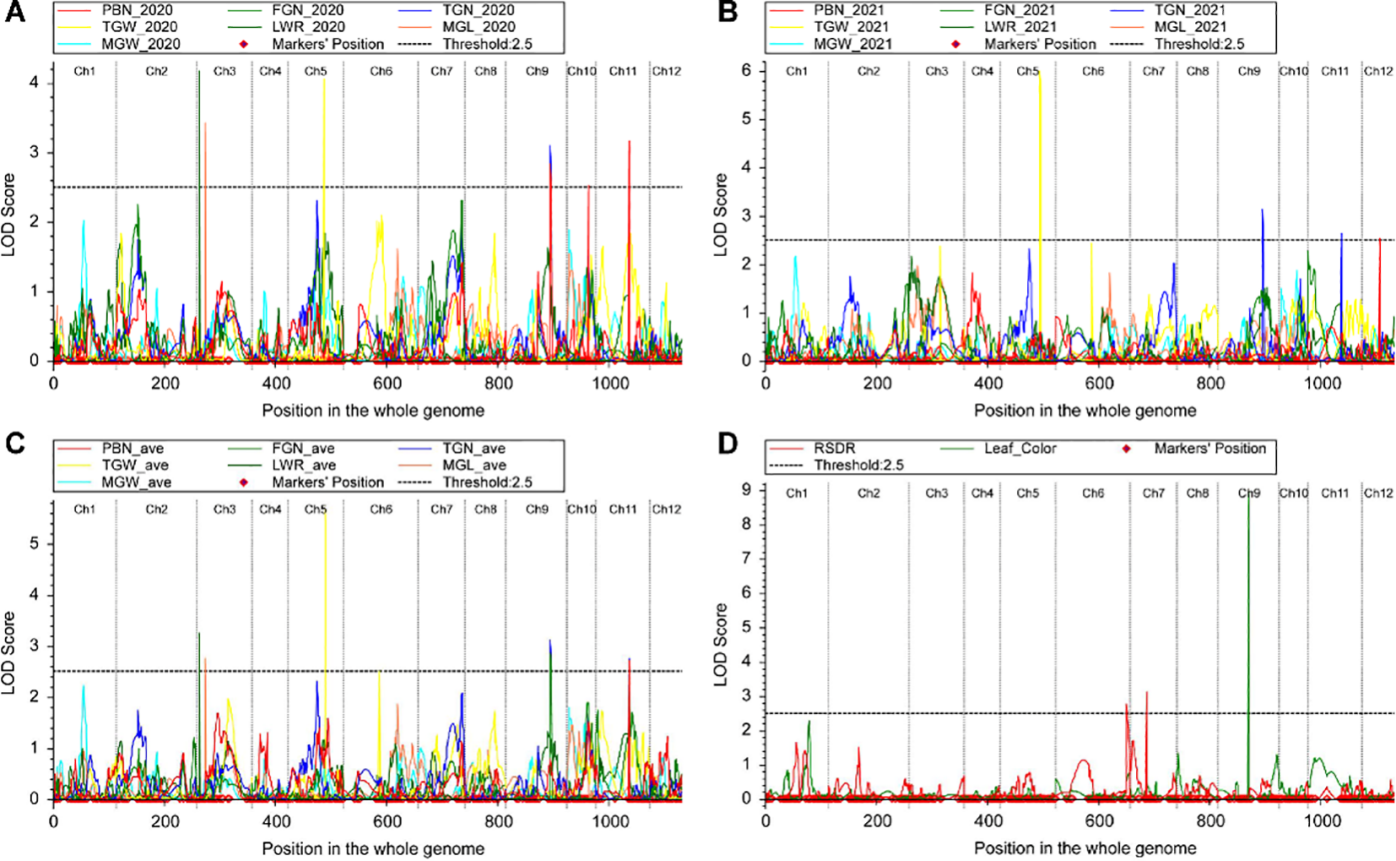
Locations and LOD of QTL for nine traits. **(A)** Distribution of QTLs for seven yield-related traits in 2020. **(B)** Distribution of QTLs for seven yield-related traits in 2021. **(C)** Distribution of QTLs for the average values of seven yield-related traits. **(D)** Distribution of QTLs for RSDR and Leaf Color.

In this study, a total of 16 QTLs associated with seven important agronomic traits were identified across five chromosomes (Chr 3, 5, 6, 7, 9, and 11) (**Table 3**, **Figures 4A-D**). These QTLs exhibited LOD (Logarithm of Odds) scores ranging from 2.52 to 8.93 and individually explained phenotypic variance (PVE) from 5.48% to 19.03%.

**Table 3:** Summary of QTL mapping for nine traits.

TraitName	QTL	Chrom	Position	LeftMarker	RightMarker	LOD	PVE(%)	LeftCI	RightCI
PBN_2020	qPBN9.1	9	80	bin1114_9	bin1113_9	2.84	5.97	79.5	82.5
PBN_2020	qPBN10.1	10	38	bin165_10	bin164_10	2.54	5.15	37.5	38.5
PBN_2020	qPBN11.1	11	61	bin241_11	bin242_11	3.17	6.52	59.5	61.5
PBN_2021	qPBN11.2	12	32	bin348_12	bin347_12	2.54	7.05	31.5	32.5
PBN_ave	qPBN11.1	11	61	bin241_11	bin242_11	2.71	6.29	59.5	61.5
FGN_2020	qFGN9.1	9	80	bin1114_9	bin1113_9	2.52	5.48	79.5	82.5
FGN_2020	qFGN11.1	11	61	bin241_11	bin242_11	2.61	5.61	59.5	61.5
FGN_ave	qFGN9.1	9	80	bin1114_9	bin1113_9	2.85	6.28	79.5	82.5
TGN_2020	qTGN9.1	9	80	bin1114_9	bin1113_9	3.11	6.50	79.5	82.5
TGN_2020	qTGN11.1	11	61	bin241_11	bin242_11	2.88	5.89	59.5	61.5
TGN_2021	qTGN9.1	9	80	bin1114_9	bin1113_9	3.14	6.63	79.5	82.5
TGN_2021	qTGN11.1	11	61	bin241_11	bin242_11	2.66	5.47	59.5	61.5
TGN_ave	qTGN9.1	9	80	bin1114_9	bin1113_9	3.13	6.57	79.5	82.5
TGN_ave	qTGN11.1	11	61	bin241_11	bin242_11	2.77	5.68	59.5	61.5
TGW_2020	qTGW5.1	5	65	bin704_5	bin703_5	4.06	8.87	64.5	65.5
TGW_2021	qTGW5.2	5	73	bin692_5	bin691_5	6.00	10.30	72.5	74.5
TGW_ave	qTGW5.3	5	68	bin698_5	bin697_5	5.68	11.47	67.5	68.5
LWR_2020	qLWR3.1	3	6	bin503_3	bin502_3	4.17	10.47	5.5	6.5
LWR_ave	qLWR3.1	3	6	bin503_3	bin502_3	3.26	8.44	5.5	6.5
MGL_2020	qMGL3.1	3	17	bin485_3	bin484_3	3.43	7.33	16.5	17.5
MGL_ave	qMGL3.1	3	17	bin485_3	bin484_3	2.77	5.86	16.5	17.5
RSDR	qRSDR6.1	6	129	bin791_6	bin790_6	2.77	6.14	127.5	132.5
RSDR	qRSDR7.1	7	29	bin910_7	bin911_7	3.11	6.82	28.5	29.5
Leaf_Color	qLeafColor9.1	9	55	bin1156_9	bin1157_9	8.93	19.03	54.5	55.5

Several QTLs for distinct panicle-related traits were mapped to overlapping genomic regions, suggesting the presence of either pleiotropic genes or tightly linked gene clusters (**Table 3**, **Figures 4A-C**). A major co-localization hotspot was identified on chromosome 9 (~80 cM), where QTLs for PBN (qPBN9.1), filled grain number (qFGN9.1), and total grain number (qTGN9.1, qTGN9.2) were consistently detected. This indicates that this genomic region harbors a key locus or loci that synergistically regulate panicle architecture and grain yield components. Another significant co-localization region was found on chromosome 11 (~61 cM), controlling qPBN11.1, qFGN11.1, and qTGN11.1, further reinforcing the genetic linkage between these traits.

The stability of QTLs across different environments or developmental stages is a critical factor for their utility in breeding programs. In this study, several QTLs demonstrated high stability by being detected in both individual years and in the analysis of mean phenotypic values. Notably, the QTLs for TGN on chromosomes 9 and 11 (qTGN9.1, qTGN9.2, and qTGN11.1) were highly stable, suggesting their genetic effects are robust against environmental fluctuations. Similarly, qPBN11.1, qFGN9.1, qLWR3.1, and qMGL3.1 were consistently identified, marking them as reliable targets for marker-assisted selection (MAS).

Additionally, we localized two salt tolerance-related QTLs (qRSDR6.1 and qRSDR7.1) using the phenotype of RSDR (**Table 3**, **Figure 4D**). Among all the QTLs identified, qLeafColor9.1 stood out as a major-effect QTL. It was mapped to chromosome 9 with an exceptionally high LOD score of 8.93 and explained a substantial 19.03% of PVE, which is considerably higher than the other QTLs (**Figure 4D**). This indicates that qLeafColor9.1 is a primary genetic determinant of leaf color variation in the studied population and represents a prime candidate for further fine-mapping and gene cloning.

### 3.5 Candidate genes predicting

Key QTLs controlling yield-related traits, namely the co-localized QTLs qPBN9.1/qFGN9.1/qTGN9.1 and qPBN11.1/qFGN11.1/qTGN11.1, were fine-mapped to two distinct genomic hotspots (**Table 4**). The first cluster, qPBN9.1/qFGN9.1/qTGN9.1, resides in a 356.50 kb region on chromosome 9 (Chr9: 12, 570, 311-12, 926, 814), while the second, qPBN11.1/qFGN11.1/qTGN11.1, spans an 809.57 kb interval on chromosome 11 (Chr11: 8, 414, 531-9, 224, 102). To elucidate the genetic basis underlying these QTLs, we conducted functional annotation and expression profiling of all genes within these intervals. Within the 356.50 kb region on chromosome 9, 49 genes were identified. Notably, five of these genes (*LOC_Os09g20900*, *LOC_Os09g20980*, *LOC_Os09g21110*, *LOC_Os09g21210*, and *LOC_Os09g20990*) exhibited specific expression in panicles or grains (**Table 5**), suggesting their direct involvement in panicle development and grain formation. In the larger 809.57 kb region on chromosome 11, 116 genes were annotated. Expression analysis revealed that only eight genes (*LOC_Os11g16280, LOC_Os11g16370, LOC_Os11g16550, LOC_Os11g14950, LOC_Os11g16410, LOC_Os11g16430, LOC_Os11g16540* and *LOC_Os11g16580*) were expressed in panicles or grains (**Table 5**, **Supplementary Figure S1**). These spatiotemporally expressed genes represent the most promising candidates for regulating panicle traits, providing clear targets for future functional validation and molecular breeding applications.

**Table 4:** Physical positions of candidate QTLs.

QTL	Chrom	Physical positions	Range	LOD	PVE(%)
qPBN9.1/qFGN9.1/qTGN9.1	9	12570311-12926814	356503	2.8431	5.9719
qPBN11.1/qFGN11.1/qTGN11.1	11	8414531-9224102	809571	3.1719	6.5223
qRSDR6.1	6	4744946-4932103	187157	2.7693	6.1387
qRSDR7.1	7	6148080-6648883	500803	3.1145	6.8204
qLeafColor9.1	9	20847909-21516552	668643	8.9339	19.0335

**Table 5:** Summry of candidate genes within QTL intervals in rice.

QTL	chromosome	start	end	gene ID	annotation
qPBN9.1/ qFGN9.1/ qTGN9.1	Chr9	12580263	12582493	LOC_Os09g20900	hypoxia-responsive family protein
	Chr9	12637640	12639373	LOC_Os09g20980	RING-H2 finger protein
	Chr9	12730527	12735642	LOC_Os09g21110	leucyl-tRNA synthetase cytoplasmic
	Chr9	12802546	12805261	LOC_Os09g21210	beta-glucan-binding protein 4
	Chr9	12644225	12650074	LOC_Os09g20990	trehalose-6-phosphate synthase
qPBN11.1/ qFGN11.1/ qTGN11.1	Chr11	8965406	8970029	LOC_Os11g16280	F-box domain containing protein
	Chr11	9034216	9042631	LOC_Os11g16370	uridine Fcytidine kinase-like 1
	Chr11	9177006	9178578	LOC_Os11g16550	uncharacterized protein ycf53
	Chr11	8417484	8422701	LOC_Os11g14950	ACT domain containing protein
	Chr11	9073778	9078878	LOC_Os11g16410	oxidoreductase short chain dehydrogenase Freductase family
	Chr11	9089498	9095181	LOC_Os11g16430	diphthamide biosynthesis protein
	Chr11	9156589	9173338	LOC_Os11g16540	tetratricopeptide repeat domain containing protein
	Chr11	9187689	9193002	LOC_Os11g16580	endonuclease III-like protein 1
qRSDR6.1	Chr6	4867843	4870168	LOC_Os06g09560	heat shock protein DnaJ
	Chr6	4897179	4900565	LOC_Os06g09630	3-oxoacyl-synthase
	Chr6	4926492	4932177	LOC_Os06g09660	auxin response factor
	Chr6	4844482	4854759	LOC_Os06g09540	SAC domain containing protein
qRSDR7.1	Chr7	6494465	6502011	LOC_Os07g11739	cytochrome P450
qLeafColor9.1	Chr9	20868717	20871108	LOC_Os09g36200 (*OsSGR*)	senescence-inducible chloroplast stay-green protein 1

**Figure 5:**
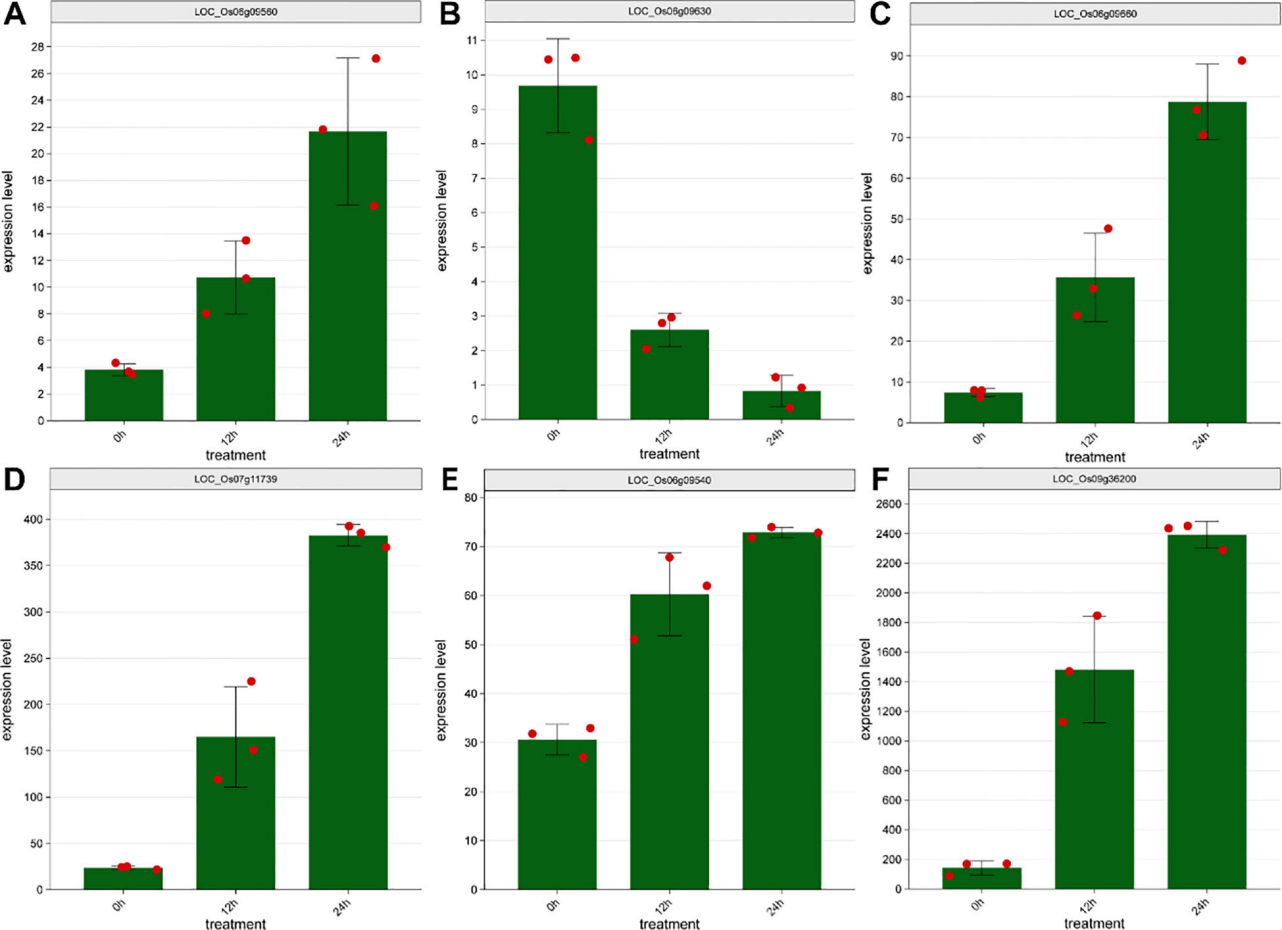
Expression of candidate genes under salt stress. **(A)***LOC_Os06g09560*. **(B)***LOC_Os06g09630*. **(C)***LOC_Os06g09660*. **(D)***LOC_Os06g09540*. **(E)***LOC_Os07g11739*. **(F)***LOC_Os09g36200* (*OsSGR*).

Two QTLs associated with salt resistance were identified based on RSDR. qRSDR6.1 was mapped to a 187.16 kb interval on chromosome 6 (Chr6:4, 744, 946-4, 932, 103), which contains 24 predicted genes. Expression profiling revealed that four of these genes, *LOC_Os06g09560*, *LOC_Os06g09630*, *LOC_Os06g09660*, and *LOC_Os06g09540*, were significantly responsive to drought stress (**Table 5** and **Figure 5**), showing either marked suppression or induction. The other QTL, qRSDR7.1, was located in a 500.80 kb region on chromosome 7 (Chr7:6, 148, 080-6, 648, 883) harboring 74 predicted genes. Notably, despite the larger number of genes within this interval, expression analysis identified only a single candidate gene that was significantly induced under drought stress: a cytochrome P450 family gene (**Table 5** and **Figure 5**).

qLeafColor9.1 is a QTL located on chromosome 9 of rice, spanning a physical interval from 20, 847, 909 bp to 21, 516, 552 bp, with a length of approximately 668.60 kb and a remarkably high LOD score of 19.03 (**Table 4**). These data strongly indicate that this locus is closely associated with leaf color variation and the senescence process. Notably, the physical position of this QTL perfectly coincides with the *OsSGR* gene (Shin et al., 2020), further corroborating the central role of *OsSGR* in regulating leaf senescence and chlorophyll degradation in rice. Given that natural variations in the promoter region of the *OsSGR* gene significantly influence the rate of senescence and grain yield, this gene can serve as a key target for molecular marker-assisted selection and gene-editing breeding strategies. Further, we observed that the expression of the *OsSGR* gene is significantly upregulated under salt stress conditions (**Figure 5**). It provides a solid theoretical foundation and valuable genetic resources for the development of novel rice varieties with delayed senescence, enhanced photosynthetic capacity, salt tolerance and increased grain yield.

## 4 Discussion

In this study, we successfully constructed a high-density genetic linkage map using a RIL population derived from a cross between Liangxiang5 and 03GY28, two rice cultivars with contrasting phenotypes in yield-related traits, salt tolerance, and leaf senescence. Through comprehensive QTL mapping, we identified 16 QTLs associated with nine key agronomic traits. Notably, we discovered major QTL hotspots controlling panicle architecture, novel QTLs for salt tolerance, and a major-effect QTL, qLeafColor9.1, which co-localizes with the *OsSGR* gene (Shin et al., 2020). We performed high-depth resequencing on the parents and identified one SNP (03GY28:C, Liangxiang5:T) in the promoter region of the *OsSGR* gene (Chr9:20867148). This finding is consistent with the results reported by Shin et al., which indicate that the T-to-C variation in the *OsSGR* promoter leads to delayed senescence, resulting in the stay-green phenotype in plants. These findings provide profound insights into the genetic basis of these complex traits and offer valuable genetic resources for future rice breeding programs.

### 4.1 Genetic dissection of yield-related traits and identification of QTL hotspots

Grain yield is a complex quantitative trait controlled by multiple genes. In this study, we identified several stable QTLs for yield components, including PBN, FGN, and TGN. A significant finding was the identification of two robust QTL hotspots on chromosomes 9 and 11, where QTLs for PBN, FGN, and TGN were consistently co-localized. The co-localization of qPBN9.1, qFGN9.1, and qTGN9.1 on chromosome 9, and qPBN11.1, qFGN11.1, and qTGN11.1 on chromosome 11, strongly suggests the presence of either pleiotropic genes or tightly linked gene clusters that synergistically regulate panicle development and grain set. This phenomenon is consistent with previous studies, which have also reported QTL clusters for yield-related traits on these chromosomes (Wei et al., 2021; Zhao X. et al., 2024; Ma Z. et al., 2025). The high stability of these QTLs across different environments (2020, 2021, and averaged data) underscores their reliability as targets for marker-assisted selection (MAS). The candidate genes identified within these intervals, such as the trehalose-6-phosphate synthase gene (*LOC_Os09g20990*) in the Chr9 hotspot, are particularly compelling. Trehalose-6-phosphate is a crucial signaling molecule in carbon metabolism and has been implicated in regulating plant development and stress responses (Li Z. et al., 2022; Li M. et al., 2024; Li J. et al., 2025), making it a strong candidate for influencing panicle traits.

### 4.2 Unraveling the genetic basis of salt tolerance

Salt stress is a major abiotic constraint to rice production (Jiang et al., 2025; Wu et al., 2025). We identified two QTLs, qRSDR6.1 and qRSDR7.1, associated with the relative salt damage rate (RSDR). The correlation analysis revealed a strong positive correlation between RSDR and grain number traits (FGN and TGN), suggesting a potential trade-off: genotypes with higher grain yield potential might be more susceptible to salt stress at the seedling stage. This intriguing finding highlights the complexity of breeding for high-yielding and salt-tolerant varieties and suggests that these traits may need to be pyramided carefully. Within the qRSDR6.1 interval, we identified four candidate genes (*LOC_Os06g09560*, *LOC_Os06g09630*, *LOC_Os06g09660*, and *LOC_Os06g09540*) that were significantly responsive to salt stress. Given the well-known crosstalk salt stress responses (often involving osmotic adjustment and oxidative stress) (Zhou et al., 2025), these genes represent promising candidates for conferring salt tolerance. Notably, *LOC_Os06g09660* encodes an auxin response factor (ARF), a key transcriptional regulator in plant growth and development. ARFs have been shown to play critical roles in modulating root system architecture (Wang C. et al., 2024; Lu et al., 2025), which is a primary determinant of water and nutrient uptake under stress conditions. The functional characterization of these genes will be crucial to validate their role in salt tolerance.

### 4.3 qLeafColor9.1: a major QTL for delayed senescence with immense breeding potential

The most striking finding of this study is the identification of qLeafColor9.1, a major-effect QTL controlling leaf color at the maturity stage. This QTL explained an exceptionally high proportion (19.03%) of the phenotypic variance, far exceeding the PVE of other QTLs identified. The physical position of qLeafColor9.1 perfectly overlaps with the *OsSGR* (STAY-GREEN) gene, a well-characterized central regulator of chlorophyll degradation and leaf senescence in rice (Shin et al., 2020). Natural allelic variation in *OsSGR* is known to be responsible for the “stay-green” phenotype, which is associated with delayed senescence, extended photosynthetic activity, and ultimately, increased grain filling and yield. Our results not only confirm the pivotal role of *OsSGR* but also provide a precise genetic location (qLeafColor9.1) and a robust marker for this valuable trait. Furthermore, our observation that *OsSGR* expression is significantly upregulated under salt stress provides a novel link between senescence regulation and abiotic stress response. This suggests that the *OsSGR* might be a key integrator of developmental and stress-induced senescence pathways. Therefore, qLeafColor9.1 represents a prime target for molecular breeding. Developing functional markers based on the causal polymorphism within *OsSGR* or using gene-editing technologies (CRISPR/Cas9) to create favorable alleles could enable the efficient development of novel rice varieties with the highly desirable “stay-green” trait, leading to enhanced photosynthetic capacity, improved stress resilience, and higher grain yield.

### 4.4 Strengths, limitations, and future perspectives

The primary strength of this study lies in the use of a high-density genetic map constructed via whole-genome resequencing, which provided high resolution and accuracy for QTL mapping. The multi-environment phenotyping over two years enhanced the reliability of the detected QTLs. However, some limitations remain. The physical intervals for the QTLs, especially the hotspots, are still relatively large (809.57 kb for the Chr11 hotspot), containing dozens of candidate genes. Fine-mapping through developing near-isogenic lines (NILs) or using a larger population is necessary to narrow down these intervals and identify the causal genes. Furthermore, while our expression analysis under salt stress provided valuable clues, the candidate genes’ functions require rigorous validation through genetic approaches, such as overexpression, RNA interference, or gene knockout.

## 5 Conclusion

In summary, this study provides a comprehensive genetic analysis of yield, salt tolerance, and senescence-related traits in rice. The identified stable QTLs, QTL hotspots, and candidate genes, especially the major-effect qLeafColor9.1 associated with *OsSGR*, serve as a solid foundation for future molecular dissection and breeding applications. The pyramiding of favorable alleles from qLeafColor9.1 (for delayed senescence), the yield-related QTL hotspots (for high yield potential), and the salt tolerance QTLs (qRSDR6.1 and qRSDR7.1) holds great promise for developing elite rice varieties capable of maintaining high productivity under suboptimal conditions, thereby contributing to global food security.

## Data Availability

The datasets presented in this study can be found in online repositories. The names of the repository/repositories and accession number(s) can be found in the article/**Supplementary Material**.

## Supplementary material

Supplementary Table 1 Phenotypic statistics of seed traits in parental.

Supplementary Table 2 Summary of whole-genome resequencing of 207 recombinant inbred lines and their parents.

Supplementary Table 3 Statistics on sequencing depth and coverage.

Supplementary Table 4 The number of Marker types.

Supplementary Table 5 The number of Marker types.

Supplementary Table 6 Statistics on genetic map gap information.

Supplementary Figure 1 Expression of 8 genes in Panicles and Seeds.
