# Supplementary material for: Integrated QTL mapping and candidate gene analysis for yield-related traits and salt tolerance in a rice RIL population
Source: Front Plant Sci. 2026 Jan 20;16:1711018. doi: 10.3389/fpls.2025.1711018 (PMC12864451; doi:10.3389/fpls.2025.1711018)
Supplement: Supplementary Table 1 — Phenotypic statistics of seed traits in parental. [file DataSheet1.docx]

Supplementary Figure 1:


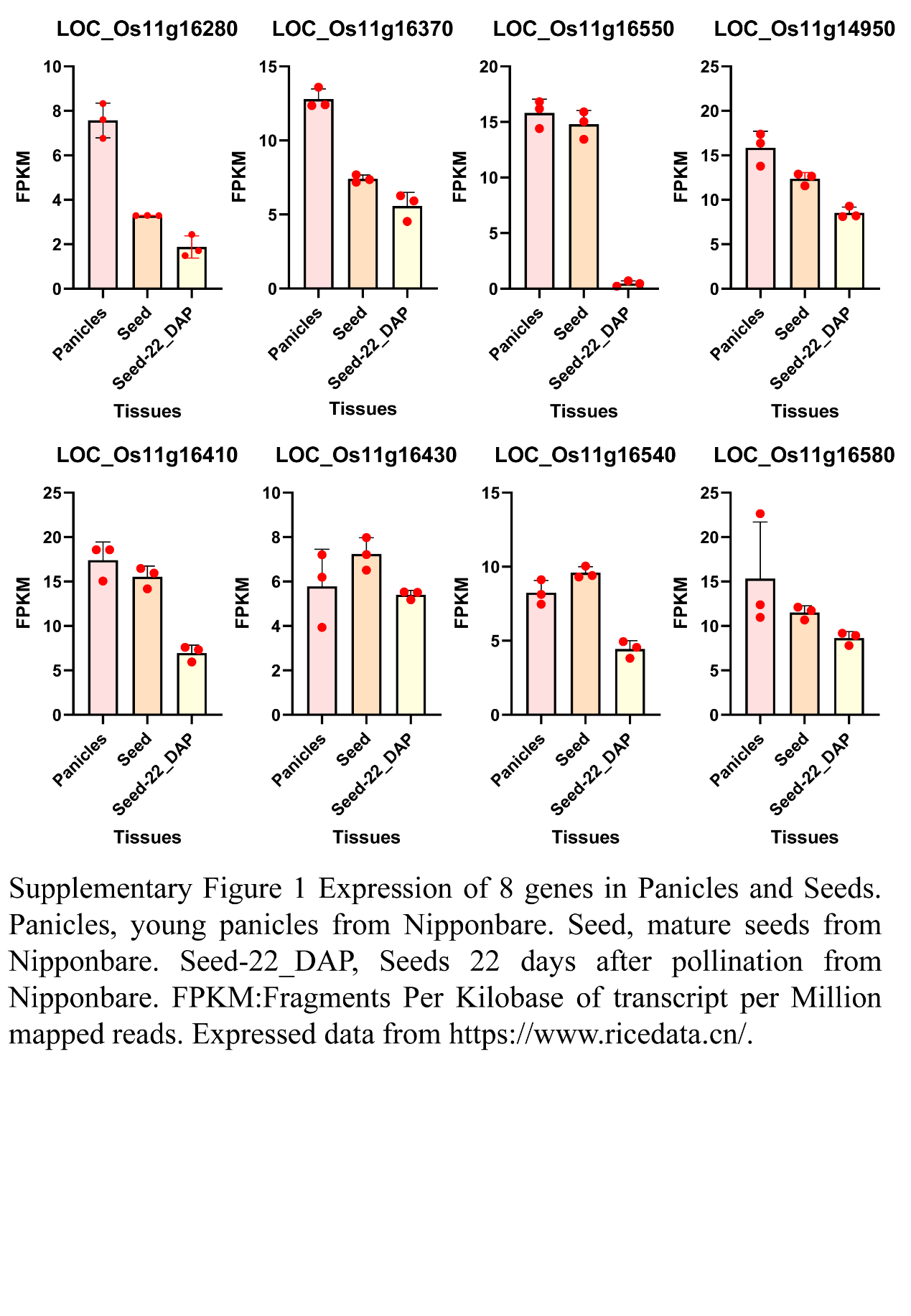


**Supplementary Figure 1** Expression of 8 genes in Panicles and Seeds.

Panicles, young panicles from Nipponbare. Seed, mature seeds from Nipponbare. Seed-22_DAP, Seeds 22 days after pollination from Nipponbare. FPKM:Fragments Per Kilobase of transcript per Million mapped reads. Expressed data from https://www.ricedata.cn/.
